# p300/Sp1-Mediated High Expression of p16 Promotes Endothelial Progenitor Cell Senescence Leading to the Occurrence of Chronic Obstructive Pulmonary Disease

**DOI:** 10.1155/2021/5599364

**Published:** 2021-08-19

**Authors:** Zhihui He, Huaihuai Peng, Min Gao, Guibin Liang, Menghao Zeng, Xuefeng Zhang

**Affiliations:** ^1^Department of Critical Care Medicine, The Third Xiangya Hospital, Central South University, Changsha, 410013 Hunan, China; ^2^Department of Critical Care Medicine, The Second Xiangya Hospital, Central South University, Changsha, 410011 Hunan, China

## Abstract

**Objective:**

Chronic obstructive pulmonary disease (COPD) is a common chronic disease and develops rapidly into a grave public health problem worldwide. However, what exactly causes the occurrence of COPD remains largely unclear. Here, we are trying to explore whether the high expression of p16 mediated by p300/Sp1 can cause chronic obstructive pulmonary disease through promoting the senescence of endothelial progenitor cells (EPCs).

**Methods:**

Peripheral blood EPCs were isolated from nonsmoking non-COPD, smoking non-COPD, and smoking COPD patients. The expressions of p16, p300, and senescence-related genes were detected by RT-PCR and Western Blot. Then, we knocked down or overexpressed Sp1 and p300 and used the ChIP assay to detect the histone H4 acetylation level in the promoter region of p16, CCK8 to detect cell proliferation, flow cytometry to detect the cell cycle, and *β*-galactosidase staining to count the proportion of senescent cells.

**Results:**

The high expression of p16 was found in peripheral blood EPCs of COPD patients; the cigarette smoke extract (CSE) led to the increase of p16. The high expression of p16 in EPCs promoted cell cycle arrest and apoptosis. The CSE-mediated high expression of p16 promoted cell senescence. The expression of p300 was increased in peripheral blood EPCs of COPD patients. Moreover, p300/Sp1 enhanced the histone H4 acetylation level in the promoter region of p16, thereby mediating the senescence of EPCs. And knockdown of p300/Sp1 could rescue CSE-mediated cell senescence.

**Conclusion:**

p300/Sp1 enhanced the histone H4 acetylation level in the p16 promoter region to mediate the senescence of EPCs.

## 1. Introduction

Chronic obstructive pulmonary disease (COPD) is a common chronic disease whose prevalence, disability rate, mortality rate, and social burden caused by it have been increasing year by year, developing into a severe public health issue. At present, it is generally believed that smoking is the major cause that induces COPD, while its pathogenesis has not been fully elucidated yet. COPD is considered to be a disease of premature lung failure [1–4]. EPCs are precursor cells of endothelial cells, which are differentiated from mesoderm angioblasts and participate in human embryonic angiogenesis [5, 6]. Due to their differentiation into endothelial cells and their biological characteristics such as secretion of vasoactive substances, proliferation, homing, and migration, endothelial progenitor cells (EPCs) play a very important role in postnatal angiogenesis, reendothelialization, tissue regeneration, and repair [7–9].

The p16 gene belongs to the INK4 gene family and consists of four members: p16*^INK4A^*, p15*^INK4B^*, p18*^INK4C^*, and p19*^INK4D^*, which all have the biological characteristics of cell growth inhibition and tumor suppression [10]. p16 is also the second most common tumor suppressor gene just after p53. It has been widely considered a familial melanoma gene, whose immunohistochemistry has a clearly defined role in certain pathological conditions [11]. Meanwhile, p16 has also been found to play a critical role in cell senescence. Cell senescence is an irreversible block of cell growth. Biochemical and morphological changes occur during cell senescence, including the formation of unique cell morphology, such as flat cytoplasm [12]. Cell senescence is an irreversible arrest of cell growth accompanied by biochemical and morphological changes, which includes the formation of unique cell morphology, such as flat cytoplasm [13]. p16-mediated senescence leads to chromatin recombination, which is associated with the inhibition of genes regulated by transcription factor E2F1 [13, 14]. Chromatin recombination in oncogene-induced premature senescence is characterized by SAHF (senescence-associated heterochromatin lesions), manifested as dense nuclear DNA and concentrated H3K9 trimethylation [15, 16].

Studies have reported that the quantity reduction and function recession of EPCs in the peripheral blood of COPD patients is highly associated with the severity of the disease [17]. In emphysema animal models, the proliferation, secretion, and adhesion of bone marrow EPCs decreased, with the expression of senescence marker p16 (INK4a) in bone marrow EPCs and lung tissues increasing, while the stem cell antigen 1 (Sca-1) and c-Kit expression decreased [18]; meanwhile, the fact that cigarette smoke extract (CSE) can directly induce the dysfunction of EPCs cultured in vitro and the changes in the expression levels of the above-mentioned gene suggest that EPC senescence and EPC gradual exhaustion exist in smoking-related COPD [18–20]. The COPD mouse model was further transplanted with allogeneic normal EPCs through the trachea, the results of which showed that after EPC transplantation, not only the lung function and emphysema pathological changes of COPD mice were significantly improved, the level and activity of matrix metalloproteinase in bronchoalveolar lavage fluid and the apoptosis of alveolar septum cells decreased, and the total antioxidant capacity increased as well [20–24]. EPCs are a very promising vascular health biomarker with broad application prospects and can be used for the treatment of a series of clinical diseases [25]. Previous studies have revealed that the quantity and function of EPCs in COPD patients might decrease, and increasing p16 expression plays an important role in maintaining characteristic cell cycle arrest [26–28]. In this study, we found high expression of p16 in the peripheral blood EPCs of COPD patients, which would lead to an increase in the transcriptional activity of p16.

Histone posttranslational modification is the main epigenetic mechanism regulating the life process, and histone acetylation is catalyzed by histone acetyltransferase [29]. Histone acetyltransferase (HAT), like p300/CBP, is a key transcriptional coactivator involved in regulating a variety of genes. Activated HAT enables p300/CBP the ability to affect chromatin activity through nucleosome histone modification. Current available data reveal that Sp1 and p300 perform cooperative work in the transcriptional regulation of several genes [30, 31]. Sp1, as a critical transcription factor in mammals, is closely associated with the formation of the Sp1/HAT complex [32, 33]. At the promoter of the eukaryote gene, Sp1 is able to recruit histone acetylase (HAT) and deacetylase (HDAC) concomitantly to regulate the histone acetylation in a dynamical and rapid way, thereby either to activate or to repress gene expressions [34–36]. Previous literature reported that p300 knockdown could reduce the expression of *β*-galactosidase (*β*-Gal) in endothelial cells and ease the senescence-like changes of endothelial cells. However, very limited researches were made upon the p300/Sp1-mediated high expression of p16 in endothelial progenitor cell senescence and the development of chronic obstructive pulmonary disease [37–39]. Therefore, in this study, we conducted a more in-depth study focusing on this regulatory mechanism.

## 2. Materials and Methods

### 2.1. Clinical Samples

This study was approved by the Third Xiangya Hospital of Central South University (No. 2018-056). We recruited three groups of subjects consisting of 18 nonsmoking non-COPD, 20 smoking non-COPD, and 20 smoking COPD patients. COPD patients were defined in accordance with the standard of the Global Initiative for Chronic Obstructive Lung Disease criteria (postbronchodilator FEV1/FVC < 0.7). COPD patients were in stable clinical state, with no evidence of respiratory infection or acute exacerbation for at least four weeks. Patients with comorbidities like asthma, interstitial lung disease, heart failure, and/or neuromuscular disease were excluded from this study (for patient information, please check Supplementary Table 1). The smoking history of subjects was determined from the mean number of pack-years of cigarette consumption. Venous blood samples (10 mL) were collected from subjects individually.

### 2.2. EPC Isolation and Identification

58 human volunteers' blood samples were successfully collected in this study. As there were 3 groups, for each group, the blood samples were collected from about 20 patients. The blood sample was collected in 4-5 batches for each group, 4 to 5 patients each batch. For each batch of collected blood samples, we mixed the blood and took 20 mL to extract EPCs for experimentation. 20 mL blood samples were diluted in EGM (Lonzo, CC-3156) in 50 mL sterile centrifuge tube (1 : 1, *v*/*v*). The same volume of diluent was added to the upper centrifuge tube containing lymphocyte separation medium (Axis-Shield). The test tube was centrifuged at 2500 rpm for 30 min at room temperature, and then, the intermediate monocyte layer was collected and placed into an empty centrifuge barrel using an aseptic suction. The cells were washed twice with PBS, and the monocytes were collected. 10% FBS or 5% pHPL and 10 U/mL heparin (Trevigen, 3450-048-08) were added to the medium in order to avoid blood platelet coagulation; then, the cells were cultured in 12-well plates coated with rat tail-derived type 1 collagen (Termo, A1048301) with EGM medium. After an overnight incubation, non-adherent cells were collected for replating. Preheated medium was added to dilute cells, and cells were isolated from 3 wells in a 12-well plate and 6 wells in a 24-well plate, performed for three times. Then, the medium was refreshed once a day for the first seven days and once every other day for the next seven days. Later on, the medium was refreshed once every 2 or 3 days. EPCs were identified through flow cytometry using CD34 (Abcam, ab64480), CD133 (Abcam, ab19898), and VEGFR2 (Abcam, ab39256) antibody. EPCs were detached, centrifuged, and washed twice with phosphate-buffered saline (PBS) and then resuspended in Stain Buffer and counted. The cell suspension was transferred to new 1.5 mL Eppendorf tubes, with roughly 5 × 10^4^ cells in each tube. 5 *μ*L of CD34 and CD133 antibodies and isotype controls was added to a 50 *μ*L cell suspension based on the concentrations of antibodies recommended in the instructions of flow cytometry. After being mixed evenly, the cell suspension containing antibodies was cultured in a refrigerator at 4°C in full darkness for 30 min, triple washed with precooled Stain Buffer, and centrifuged for 5 min at 400 g. The unbound antibodies were washed away. In the end, cells were resuspended in flow tubes with 500 *μ*L Stain Buffer and detected by flow cytometry. The results of flow cytometry were analyzed and processed by using FlowJo 7.6 software.

### 2.3. Western Blot

The cells were washed three times with precooled PBS, lysed with RIPA lysate, and centrifuged at 12000 rpm for 10 min; then, the supernatant was gathered for detecting protein concentration using a BCA detection reagent (Beijing Kangwei Century Biotechnology Co., Ltd., CW0014). After electrophoresis with voltage altered from 60 V to 120 V, the protein was transferred to the PVDF membrane using wet-to-electric transfer. Following that, the membrane was blocked using 5% skim milk-TBST and cultured overnight at 4°C with primary antibodies as follows: anti-Col1a1 (CST, 84336; 1 : 1000), MMP3 (Abcam, ab52915; 1 : 1000), MMP13 (Abcam, ab39012; 1 : 1000), Pal1 (Abcam, ab7205; 1 : 1000), and GAPDH (Thermo, AM4300; 1 : 5000). Subsequently, the membrane was cultured with horseradish peroxidase-labeled goat anti-rabbit IgG (Beijing CoWin Biosciences, China). Afterwards, Tanon™ High-sig ECL Western Blotting Substrate (Shanghai Tanon Co., Ltd., 180-501) was used to develop the film, and the gray value was detected by ImageJ (NIH). Thereafter, the protein level was expressed by the ratio of the gray value of the target bands to that of the internal reference (GAPDH).

### 2.4. RNA Extraction and RT-PCR

Total RNA was extracted by TRIzol (Thermo Fisher, 15596026) and reverse-transcribed into cDNA by PCR amplification instrument (Bio-Rad). Subsequently, real-time quantitative RT-PCR experiments were conducted using ABI 7500 quantitative PCR instrument (ABI 7500, Thermo Fisher), with reaction conditions set as follows: predenaturation at 95°C for 10 min, 40-cycle denaturation at 95°C for 10 s, annealing at 60°C for 20 s, and extension at 72°C for 34 s. Then, the samples were analyzed using either PCR or quantitative PCR. The primer pairs of p16 were as follows: sense 5′-TTCCTGGACACGCTGGT-3′ and antisense 5′-CAATCGGGGATGTCTGAG-3′. The primer pairs of p300 were as follows: sense 5′-GACCCTCAGCTTTTAGGAATCC-3′ and antisense 5′-TGCCGTAGCAACACAGTGTCT-3′. The primer pairs of *β*-actin were as follows: sense 5′-TCGTGCGTGACATTAAGGAG-3′ and antisense 5′-ATGCCAGGGTACATGGTGGT-3′. The primer pairs of Col1a1 were as follows: sense 5′-GCAGCTGGGTCCTCAGAAT-3′ and antisense 5′-CAGTTCCCCAGTTCCACTTC-3′. The primer pairs of MMP3 were as follows: sense 5′-CAGACTTGTCCCGTTTCCAT-3′ and antisense 5′-GGTGCTGACTGCATCAAAGA-3′. The primer pairs of MMP13 were as follows: sense 5′-CAGACTTGTCCCGTTTCCAT-3′ and antisense 5′-GGTGCTGACTGCATCAAAGA-3′. The primer pairs of Pal1 were as follows: sense 5′-TCTACAACAACGGATTGCCGTCC-3′ and antisense 5′-CACGGTGTTCTTCACCGCGTGC-3′. Data were analyzed using the 2^−ΔΔCt^ method with *β*-actin acting as the internal control.

### 2.5. Cell Cycle Analysis by Flow Cytometry

Cells were digested with trypsin, fixed with 70% ethanol, preserved and stored overnight at 4°C, then suspended in 50 *μ*L phosphate buffer with 0.5 *μ*L and 10 *μ*g/mL RNase A and 150 *μ*L propidium iodide. Following that, cell DNA content was quantified using FACSCalibur (BD Biosciences, San Jose, CA, USA).

### 2.6. Chromatin Immunoprecipitation

ChIP protocol has been described previously [40] with antiacetyl H4 (Millipore, 06-866) antibody used. The samples were assessed by either PCR or RT-QPCR.

### 2.7. Cell Transfection

Before cell transfection, EPCs were seeded into 24-well plates, and 0.5 mL contained approximately 1 × 10^5^ cells. Plasmid transfection was performed using Lipofectamine™ 2000 (Thermo Fisher, 1668030) based on the Lipofectamine 2000 transfection instructions. RNAi Max (Invitrogen, 13778075) was transfected with 50 nmol/L siRNA in serum-free and antibiotic-free medium according to the manufacturer's protocol. The siRNA sequences were as follows: Sp1 siRNA 1: CCUGGAGUGAUGCCUAAUATT; Sp1 siRNA 2: CCAGCAACAUGGGAAUUAUTT; p300 siRNA 1: GCAGCUCAACCAUCCACUATT; p300 siRNA 2: GCAAACAAUCGAGCGGAAUTT; and p16 siRNA: AGAACCAGAGAGGCTCTGA.

The medium was changed to the complete growth condition 6 hours after transfection; then, the cells were harvested 72 hours after transfection.

### 2.8. Immunofluorescence

The cells were washed twice using PBS (5 min each), fixed with 4% paraformaldehyde for 15 min, incubated with 0.5% Triton/PBS for 6 min, blocked (10% goat serum, 0.05% NaN_3_, 0.2% Triton, and diluted with PBS at 37°C for 30 min), and incubated with primary antibody overnight at 4°C. Following that, the cells were incubated with a fluorescent secondary antibody (Thermo Fisher) in full darkness for 1 hour, stained with DAPI for 5 min and sealed. Finally, a confocal microscope (Leica SP5) was used to take pictures of the cells, or a fluorescence microscope was used to observe the cells.

### 2.9. *β*-Gal Staining

Basing on the manufacturer's protocol, cells were stained with SA-*β*-gal activity using a cell senescence detection kit (Millipore, KAA002). Then, positive staining was quantified using ImageJ and Image Pro Premier software.

### 2.10. Cell Counting Kit-8 (CCK8) Assay

Each plate was inoculated with cells basing on the experimental group set. The cells in the logarithmic growth phase were made into cell suspension, and the inoculation density was set as 3 × 10^4^ cells/mL, and 100 *μ*L cell suspension was placed in a 96-well plate with three replicate wells which were inoculated in each group. 100 *μ*L of culture medium was used as blank control and incubated at 37°C overnight in a 5% CO_2_ incubator. A Cell Counting Kit-8 (CCK8) and serum-free DMEM were mixed with a volumetric ratio of 1 : 10 and added to the test wells at a dose of 100 *μ*L/well, then incubated at 37° C for 1 h in a 5% CO_2_ incubator. The absorbance at 450 nm was measured using a microplate reader, with the plate values recorded.

### 2.11. Luciferase Experiment

p300 and Sp1 were cotransfected with the luciferase vector driven by a p16 promoter. The 505 bp fragment of p16 was amplified by PCR with p16F 5′-CCAAACAC-CCCGATTCAATTTGGCA-3′ and p16R5′-CCGCTGCCTGCTCTACCCCTCTCC-3′ primers to produce the luciferase reporter plasmid of p16 promoter. The PCR fragment was cloned into PCR 2.1-TOPO vector, and the sequence was verified. After 48 h of transfection, the Luciferase Reporter Assay System (Thermo) was used for detection, and the specific steps were in accordance with the corresponding kit instructions. The cell culture medium in each hole was discarded, and 100 *μ*L 1 × cell lysate was added to each hole. The cell lysate was oscillated on a shaker for 30 min, and the impurities were precipitated by centrifugation (1200 rpm, 1 min). A total of 20 *μ*L of cell lysate was added to each well of the opaque 96-well plate, and 100 *μ*L of luciferase detection reagent II (LAR II) and 100 *μ*L of sea kidney luciferase reagent were added in turn according to the instructions. Tecan Infinite F200/M200 luciferase activity value (*R*) of each well was detected by Tecan Infinite F200/M200 multifunctional microplate reader, and the *F*/*R* value was used as the relative activity value of each well for statistical analysis.

### 2.12. Statistical Analysis

All the data were presented as mean ± standard deviation. The data of two groups were compared with a *T* test, and the data of multiple groups were analyzed using one-way analysis of variance (ANOVA). Data analysis was conducted using SPSS17.0 (SPSS, Inc., Chicago, Illinois, USA) and GraphPad Prism 8.0 (GraphPad Software, San Diego, California, USA). Statistical significance was assumed when *P* < 0.05 while significantly difference was confirmed when *P* < 0.01.

## 3. Results

### 3.1. High Expression of p16 Occurred in Peripheral Blood EPCs of COPD Patients

**Figure 1:**
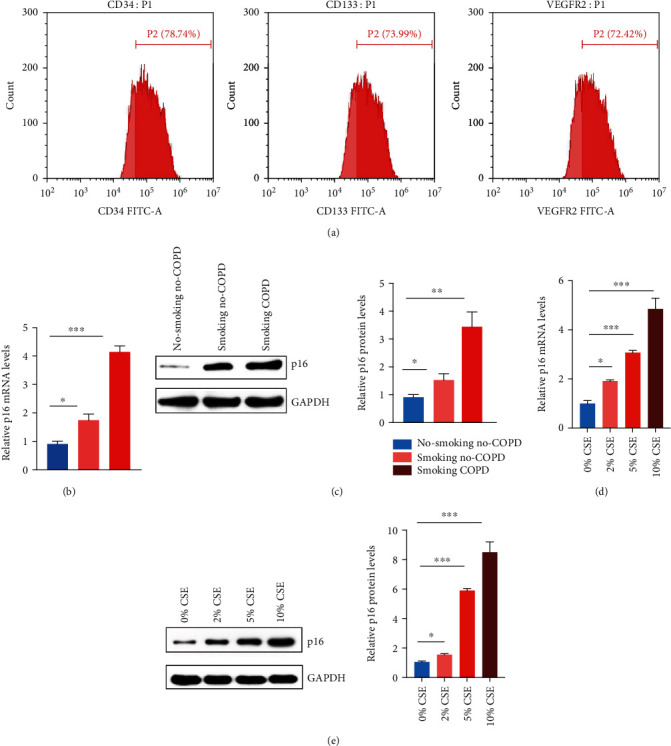
High expression of p16 in EPCs of COPD patients. (a) Peripheral blood EPCs were isolated from nonsmoking non-COPD patients. Flow cytometry verified that the isolated cells were peripheral blood endothelial cells. (b) RT-PCR was used to detect the expression of p16 in peripheral blood EPCs of nonsmoking non-COPD, smoking non-COPD, and smoking COPD patients. (c) Western Blot was used to detect the expression of p16 in peripheral blood EPCs of nonsmoking non-COPD, smoking non-COPD, and smoking COPD patients setting each group with 20 samples. (d) Peripheral blood EPCs from nonsmoking non-COPD patients were treated with different concentrations of CSE, and the expression of p16 was detected by RT-PCR. (e) Peripheral blood EPCs from nonsmoking non-COPD patients were treated with different concentrations of CSE, and the expression of p16 was detected by Western Blot. ^∗^*p* < 0.05, ^∗∗^*p* < 0.01, and ^∗∗∗^*p* < 0.001.

As a fundamental cytokine, p16 can regulate the cell senescence process. In order to explore the expression of p16 in peripheral blood EPCs of nonsmoking non-COPD, smoking non-COPD, and smoking COPD patients, we firstly isolated peripheral blood EPCs of these patients and identified those isolated cells by flow cytometry (Figure 1(a)). Then, we detected the expression of p16 in different groups by RT-PCR and Western Blot, whose results showed that the mRNA and protein levels of p16 were significantly increased in peripheral blood EPCs of COPD patients (Figures 1(b) and 1(c)). Then, we further verified whether CSE would lead to the increase of p16. We cultured EPCs from the peripheral blood of nonsmoking and non-COPD patients with different concentrations of CSE. RT-PCR results showed that the expression of p16 increased gradually with the increase of CSE concentration. Meanwhile, Western Blot results showed that the protein level of p16 increased significantly with the increase of CSE concentration (Figures 1(d) and 1(e)).

### 3.2. High Expression of p16 in EPCs Inhibited Cell Activity and Promoted Cell Cycle Arrest

**Figure 2:**
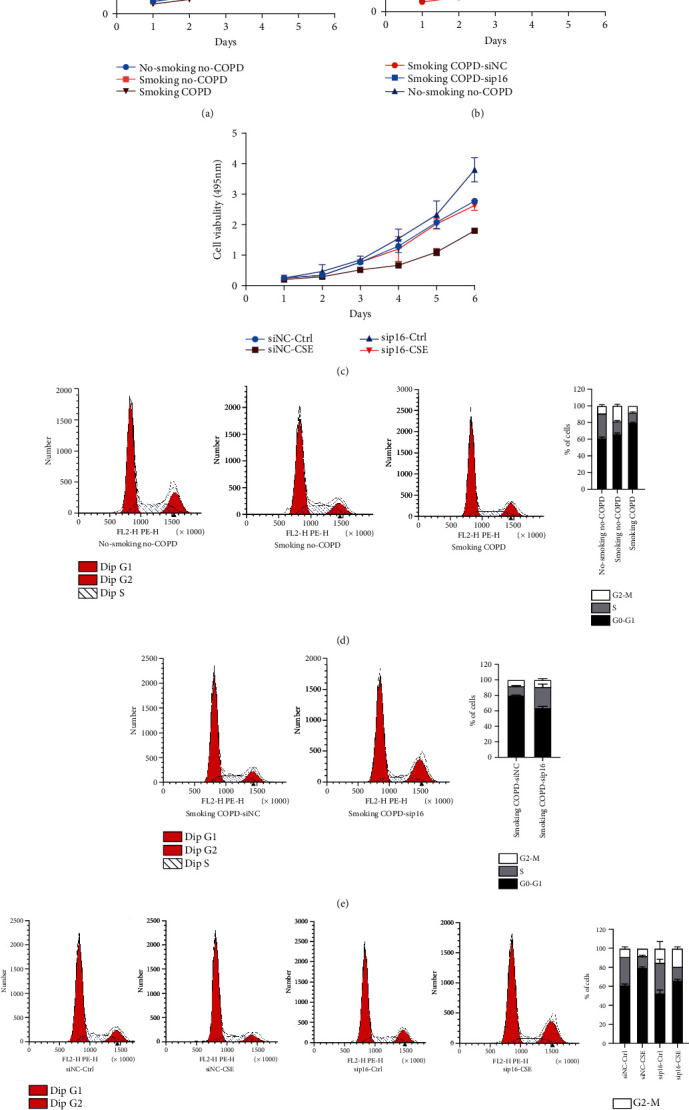
High expression of p16 in EPCs promoted cell cycle arrest and apoptosis. (a) Peripheral blood EPCs were isolated from nonsmoking non-COPD, smoking non-COPD, and smoking COPD patients, and cell proliferation was detected by CCK8. (b) Peripheral blood EPCs of smoking COPD patients were isolated, p16 was knocked down by siRNA, and cell proliferation was detected by CCK8. (c) Peripheral blood EPCs from nonsmoking non-COPD were treated with 5% CSE, p16 was knocked down by siRNA, and cell proliferation was detected by CCK8. (d) Peripheral blood EPCs were isolated from nonsmoking non-COPD, smoking non-COPD, and smoking COPD patients, and cell cycle was detected by flow cytometry. (e) Peripheral blood EPCs from smoking COPD patients were isolated, siRNA knocked down p16, and cell cycle was detected by flow cytometry. (f) Peripheral blood EPCs from nonsmoking non-COPD patients were treated with 5% CSE, p16 was knocked down by siRNA, and cell cycle was detected by flow cytometry.

We then detected the proliferation of EPCs in nonsmoking non-COPD, smoking non-COPD, and smoking COPD patients. The CCK8 assay showed that the proliferation ability of EPCs in smoking COPD patients decreased markedly (Figure 2(a)). Further, we knocked down p16 in EPCs of smoking COPD patients and found that the low expression of p16 could rescue the decrease of endothelial progenitor cell activity in those patients (Figure 2(b)). While the CSE treatment was conducted, we found during the meantime that knocking down the expression of p16 in EPCs could effectively block the inhibitory effect of CSE on cell proliferation (Figure 2(c)). We further analyzed the cell cycle of EPCs in nonsmoking non-COPD, smoking non-COPD, and smoking COPD patients, the results of which showed that G1/S phase transition arrest occurred in the EPCs of all the smoking COPD patients (Figure 2(d)). Moreover, we knocked down p16 in EPCs of smoking COPD patients, finding that the low expression of p16 could rescue the G1/S phase arrest of EPCs in those patients (Figure 2(e)). At the same time, we knocked down p16 in CSE-treated non-COPD EPC cells, and we found that knocking down p16 could inhibit the G1/S phase arrest caused by CSE (Figure 2(f)).

### 3.3. CSE-Mediated p16 Overexpression Promoted Cell Senescence

**Figure 3:**
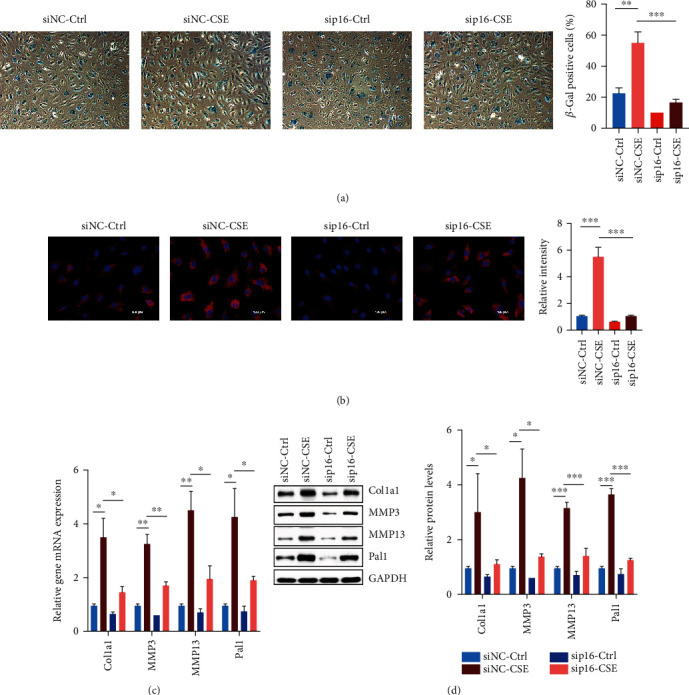
CSE-mediated high expression of p16 promoted cell senescence. (a) Peripheral blood EPCs from nonsmoking non-COPD patients were treated with 5% CSE, p16 was knocked down by siRNA, and the proportion of senescence cells was counted by *β*-galactosidase staining. (b) Peripheral blood EPCs from nonsmoking non-COPD patients were treated with 5% CSE, p16 was knocked down by siRNA, and the expression of Lamp1 was detected by immunofluorescence. (c) Peripheral blood EPCs from nonsmoking non-COPD patients were treated with 5% CSE, p16 was knocked down by siRNA, and the expressions of senescence-related genes Col1a1, MMP3, MMP13, and Pal1 were detected by RT-PCR. (d) Peripheral blood EPCs from nonsmoking non-COPD patients were treated with 5% CSE, p16 was knocked down by siRNA, and the expressions of senescence-related genes Col1a1, MMP3, MMP13, and Pal1 were tested by Western Blot. ^∗^*p* < 0.05, ^∗∗^*p* < 0.01, and ^∗∗∗^*p* < 0.001.

To investigate the effect of the high expression of p16 on senescence of endothelial progenitor cells, we performed *β*-Gal staining and detected the expression of senescence-related genes. The *β*-Gal staining experiment revealed that CSE could promote the senescence of EPCs. We also found that knocking down the expression of p16 in EPCs could inhibit the cell senescence caused by CSE (Figure 3(a)). Immunofluorescence staining demonstrated that CSE promoted the high expression of Lamp1, which indicated the increase of lysosomes, the high autophagy of cells, and increasing senescence degree of EPCs, while knocking down p16 would reduce the expression of Lamp1 to a certain degree (Figure 3(b)). Meantime, we also detected the expression of senescence-related genes. Senescence-related genes Col1a1, MMP3, MMP13, and Pal1 were highly expressed in EPCs treated with CSE, while the expressions of those genes were inhibited with p16 knocked down (Figures 3(c) and 3(d)).

### 3.4. Increased Expression of p300 Promoted the High Expression of p16

**Figure 4:**
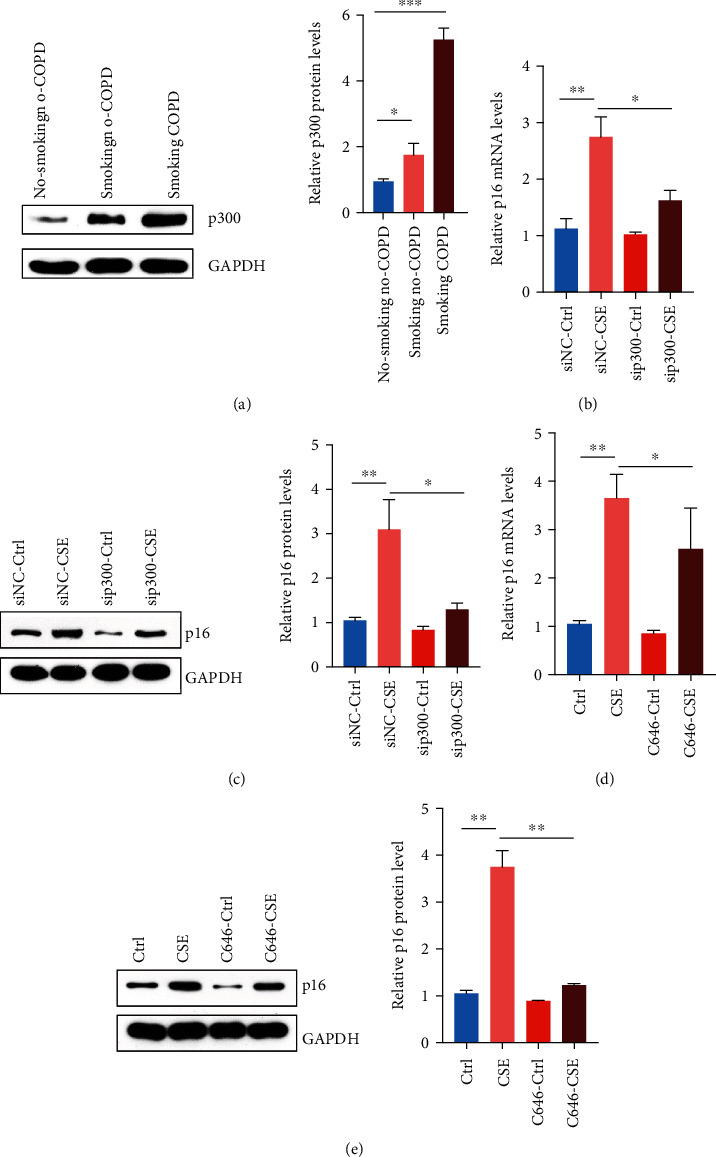
Increased p300 mediated the high expression of p16. (a) Western Blot was used to detect the expression of p300 in peripheral blood EPCs of nonsmoking non-COPD, smoking non-COPD, and smoking COPD patients setting each group with 20 samples. (b) Peripheral blood EPCs from nonsmoking non-COPD patients were treated with 5% CSE, p300 was knocked down by siRNA, and the expression of p16 was detected by RT-PCR. (c) Peripheral blood EPCs from nonsmoking non-COPD patients were treated with 5% CSE, p300 was knocked down by siRNA, and the expression of p16 was tested by Western Blot. (d) Peripheral blood EPCs from nonsmoking non-COPD patients were treated with 5% CSE and p300 small molecule inhibitor C646, and the expression of p16 was detected by RT-PCR. (e) Peripheral blood EPCs from nonsmoking non-COPD patients were treated with 5% CSE and p300 small molecule inhibitor C646, and the expression of p16 was tested by Western Blot. ^∗^*p* < 0.05, ^∗∗^*p* < 0.01, and ^∗∗∗^*p* < 0.001.

Previous studies reported that p300 could regulate the transcriptional activity of p16, and the high expression of p300 could promote cell cycle arrest [41]. We also discovered that among nonsmoking non-COPD, smoking non-COPD, and smoking COPD patients, the expression of p300 in the peripheral blood progenitor cells of smoking COPD patients was remarkably higher than that of the other two groups (Figure 4(a)). When the cells were treated with CSE and the expression of p300 was knocked down, the expression of p16 was also decreased as revealed by RT-PCR and Western Blot results (Figures 4(b) and 4(c)). When the small molecule inhibitor of p300 (C646) was added to the EPCs treated with CSE, we found that the decrease of p300 activity could inhibit the expression of p16 to some extent. All those together suggest that in the high expression of p16 mediated by CSE, p300 is very likely to be a potential transcriptional regulator in the upstream of p16.

### 3.5. p300/Sp1 Regulated p16 Transcriptional Activity

**Figure 5:**
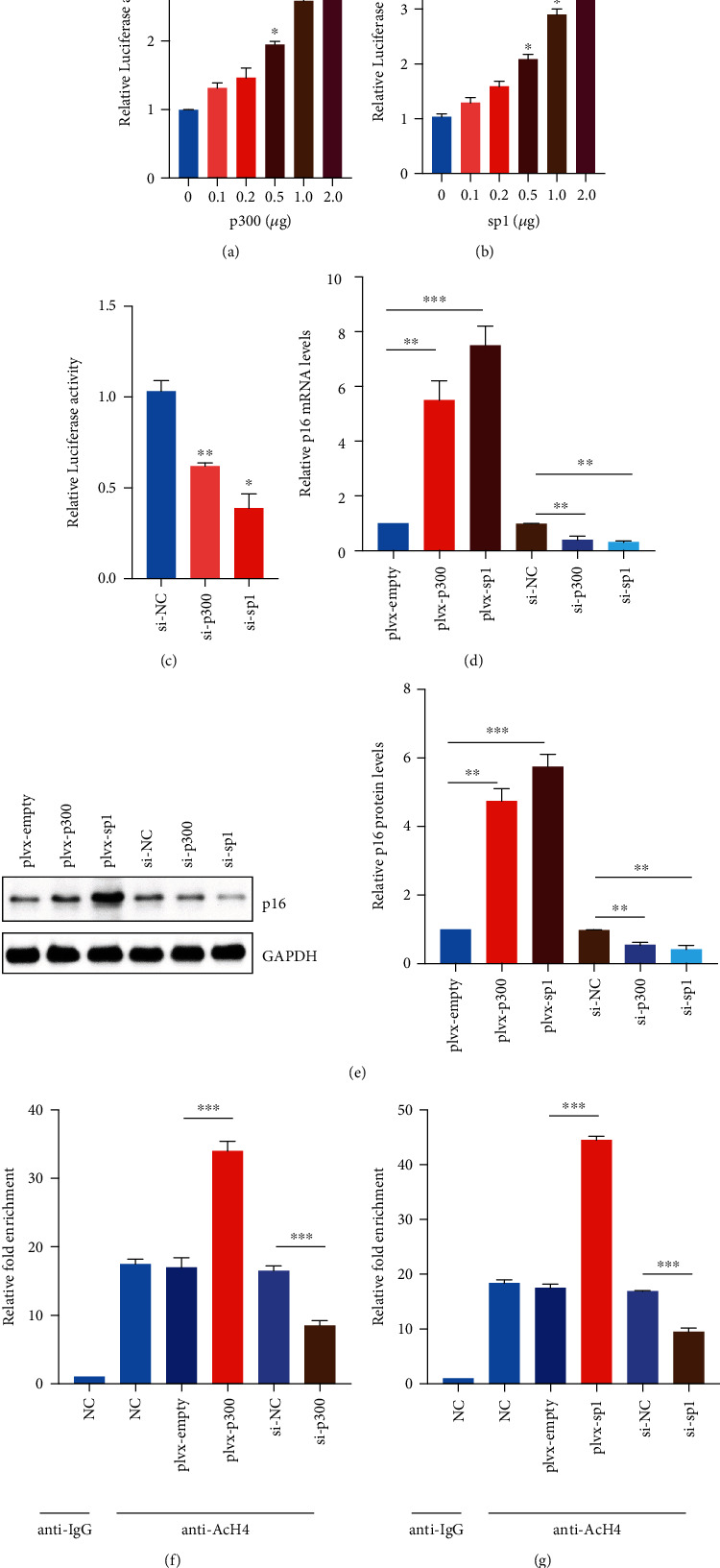
p300/Sp1 regulated p16 transcriptional activity. (a) Different amounts of p300 were overexpressed in EPCs, and the activity of the p16 promoter was detected by luciferase. (b) Different amounts of Sp1 were overexpressed in EPCs, and the activity of the p16 promoter was detected by luciferase. (c) p300/Sp1 were knocked down in EPCs, and the activity of the p16 promoter was detected by luciferase. (d) p300/Sp1 were overexpressed or knocked down in EPCs, and the expression of p16 was detected by RT-PCR. (e) p300/Sp1 were overexpressed or/and knocked down in EPCs, and the expression of p16 was tested by Western Blot. (f) p300 was overexpressed or/and knocked down in EPCs, and the level of histone H4 acetylation in the p16 promoter region was detected by ChIP assay. (g) Sp1 was overexpressed or/and knocked down in EPCs, and the level of histone H4 acetylation in the p16 promoter region was detected by the ChIP assay. All the EPCs used in this figure were from nonsmoking non-COPD patients. ^∗^*p* < 0.05, ^∗∗^*p* < 0.01, and ^∗∗∗^*p* < 0.001.

Previous studies suggested that p300 functions as a transcriptional coactivator to regulate many cellular responses such as cell cycle progression and cellular differentiation, and this process relies on the transcriptional factor Sp1. Meanwhile, Sp1 directs the formation of preinitiation complexes to -464 to -452 bp region of the p16 promoter [41, 42]. In order to validate whether this signaling pathway could also regulate the senescence of EPCs mediated by CSE, we firstly overexpressed different amounts of p300 in EPCs and detected the activity of the p16 promoter by luciferase assay. We found that p16 promoter activity increased with the increase of p300 expression (Figure 5(a)). Overexpression of Sp1 could also promote the transcriptional activity of the p16 promoter (Figure 5(b)), while knockdown of p300 or Sp1 would inhibit the transcriptional activity of the p16 promoter (Figure 5(c)). We overexpressed or knocked down p300/Sp1 in EPCs and found that overexpression of p300/Sp1 promoted the high expression of p16 in EPCs, while knockdown of p300/Sp1 inhibited the expression of p16 (Figures 5(d) and 5(e)). ChIP assay displayed that overexpression of p300/Sp1 promoted histone H4 acetylation in the p16 promoter region, while knockdown of these two genes had the opposite effect (Figures 5(f) and 5(g)). The above results indicated that p300/Sp1 were jointly involved in the high expression of p16 in EPCs mediated by CSE.

### 3.6. Low Expression of p300/Sp1 Inhibited CSE-Mediated Cell Senescence

**Figure 6:**
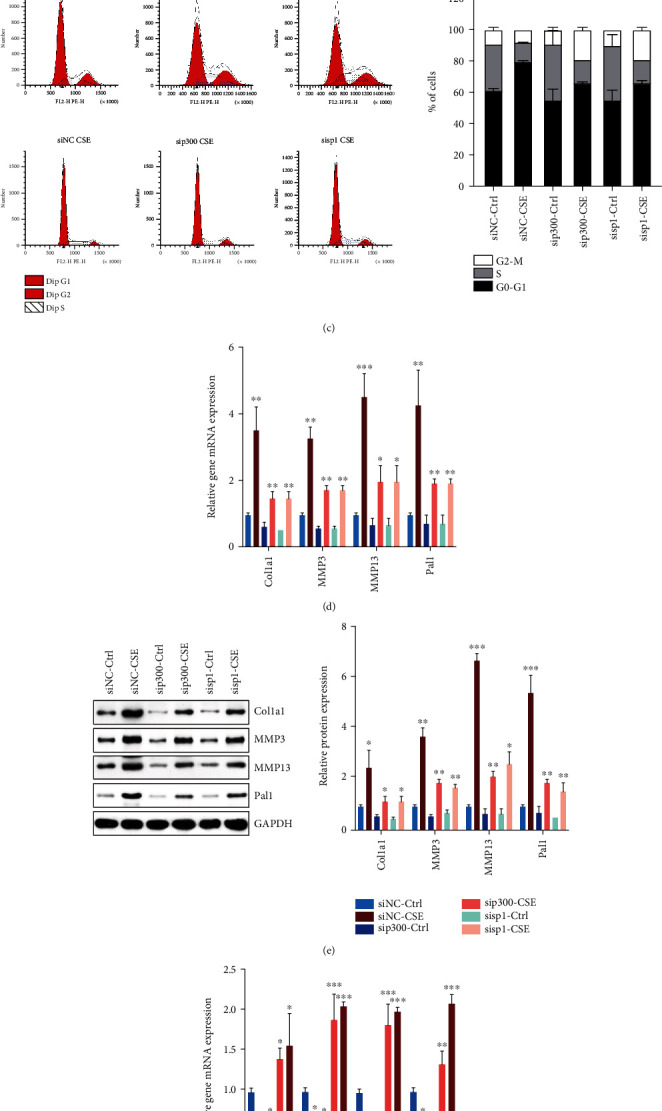
Low expression of p300/Sp1 inhibited CSE-mediated cell senescence. (a) Peripheral blood EPCs were treated with 5% CSE, p300/Sp1 were knocked down by siRNA, and the proportion of senescence cells was counted by *β*-galactosidase staining. (b) Peripheral blood EPCs were treated with 5% CSE, p300/Sp1 were knocked down by siRNA, and the expression of Lamp1 expression was detected by immunofluorescence. (c) Peripheral blood EPCs were treated with 5% CSE, p300/Sp1 were knocked down by siRNA, and cell cycle distribution was detected by flow cytometry. (d) Peripheral blood EPCs were treated with 5% CSE, p300/Sp1 were knocked down by siRNA, and the expressions of senescence-related genes Col1a1, MMP3, MMP13, and Pal1 were detected by RT-PCR. (e) Peripheral blood EPCs were treated with 5% CSE, p300/Sp1 were knocked down by siRNA, and the expressions of senescence-related genes Col1a1, MMP3, MMP13, and Pal1 were tested by Western Blot. (f) Peripheral blood EPCs were treated with 5% CSE, p300/Sp1 were knocked down by siRNA, p16 was overexpressed, and the expressions of senescence-related genes Col1a1, MMP3, MMP13, and Pal1 were detected by RT-PCR. (g) Peripheral blood EPCs were treated with 5% CSE, p300/Sp1 were knocked down by siRNA, p16 was overexpressed, and the expressions of senescence-related genes Col1a1, MMP3, MMP13, and Pal1 were tested by Western Blot. All the EPCs used in this figure were from nonsmoking non-COPD patients. ^∗^*p* < 0.05, ^∗∗^*p* < 0.01, and ^∗∗∗^*p* < 0.001.

To further testify that p300/Sp1 regulates the high expression of p16 in EPCs mediated by CSE, we knocked down p300 or Sp1 in EPCs treated with CSEs. Both *β*-Gal staining and Lamp1 immunofluorescence experiments demonstrated that knocking down p300 or Sp1 could inhibit cell senescence mediated by CSE to a certain extent (Figures 6(a) and 6(b)). Similarly, flow cytometry results also showed that knocking down p300 or Sp1 inhibited the cell cycle arrest of EPCs (Figure 6(c)). At the same time, we also detected the expression of senescence-related genes Col1a1, MMP3, MMP13, and Pal1, finding that knockdown of p300 or Sp1 could also inhibit the CSE-mediated high expression of those senescence genes to a certain degree (Figures 6(d) and 6(e)). Further, we also performed the rescue experiment. We knocked down p300 or Sp1 in CSE-treated EPCs and overexpressed p16. We found that after overexpressing p16, the mRNA and protein levels of senescence-related genes Col1a1, MMP3, MMP13, and Pal1 were significantly increased, indicating that CSE regulated the expression of p16 through p300 or Sp1, thereby causing the senescence of EPCs (Figures 6(f) and 6(g)).

## 4. Discussion

COPD is the most common respiratory system disease and the fourth leading cause of death all over the world. It is featured by progressive airflow obstruction, which is a response to harmful particles or gases, especially cigarette smoke, and is related to the chronic inflammatory process of airway and lung parenchyma [43]. The pathogenic mechanism of COPD has not been fully uncovered yet. Our study found that p16 expression increased in EPCs of COPD patients, which inhibited cell activity, promoted cell cycle arrest, and enhanced senescence of vascular endothelial cells. Vascular EPCs are important biomarkers of vascular health. Previous studies have indicated that there exists a certain degree of vascular EPC reduction in COPD patients. Our study suggested that decreased EPCs were highly likely to be caused by increased p16 expression induced by smoking. Furthermore, we found that CSE increased the expression of p300/sp1, which in turn mediated the increase of p16 expression in EPCs. Importantly, our study demonstrated that CSE truly promoted the progress of COPD, providing a theoretical basis for explaining the pathogenesis of COPD.

Histone acetyltransferase p300 is a transcription activator, which was originally discovered in the search for the adenovirus carcinogenic transcription factor E1A binding protein, and later, it was demonstrated to have histone acetyltransferase activity [44, 45]. Both the lung tissues of mice exposed to cigarette smoke and the human bronchial epithelial cells induced by CSE display an upregulation of the histone H4K12 acetylation level. Knockdown of p300 can reduce the expression of *β*-galactosidase and slow down the senescence-like modifications of endothelial cells [46]. In this study, we found that p300 upregulated the expression of p16. The increase of histone acetyltransferase p300 in COPD may be involved in the pathogenesis of COPD by upregulating the acetylation level of histone H4K12, thereby activating the expression of senescence-related factors and promoting the senescence of EPCs. Since p300 is a transcription activator, it is also worth studying whether p300 will activate other transcription factors in addition to p16. Meanwhile, we did not know exactly why there existed a high expression of p300 in peripheral blood EPCs of COPD patients.

Histone modifying enzymes are regulated by the ubiquitin-proteasome degradation pathway. Ubiquitination modification is regarded as the signal of protein degradation [47]. And in cells, the ubiquitin-proteasome pathway is the main pathway for ATP-dependent protein-selective degradation, which mainly functions on some regulatory proteins with short half-life and some structural abnormalities, misconfigurations, or damaged proteins in cells to regulate cell activities [48, 49]. Rom et al. [50] found that cigarette smoking exposure could activate the ubiquitin-proteasome pathway, leading to skeletal muscle protein degradation and cell damage. Jeong et al. [51] reported that the activated ubiquitin-proteasome pathway in lung cancer cells could result in the degradation of histone acetyltransferase p300. It is also worth studying whether there exists a smoking-induced deubiquitinase activation in the body that could promote the senescence of EPCs to participate in the process of COPD through stabilizing the expression of p300 protein, upregulating the acetylation level of histone H4K12, and activating the transcription of senescence-related factors.

The study limitations mainly lie on the limited patient population covered by the analysis. The COPD patients selected in this study were mainly acute exacerbations including no stable patients. Meanwhile, since we studied endothelial progenitor cells extracted from the peripheral blood of multiple patients and did not specifically compare the activity of p300/p21 in endothelial progenitor cells of patients with grade A-D COPD, our study could not fully represent the entire clinical spectrum of COPD patients.

It has been reported that in patients with early COPD, the number of EPCs increases, and EPCs contribute to the repair and reconstruction of pulmonary vessels, while in patients with late COPD, the number of EPCs in circulation decreases [52–54]. In this study, we did not explore whether the number and function of circulating EPCs in four patients changed. Follow-up studies will explore the above issues more in-depth.

In general, our study found increased expressions of p16 and p300 in peripheral blood EPCs of COPD patients, and CSE would lead to the increase of p16. In addition, p300/Sp1 enhanced the histone H4 acetylation level in the p16 promoter region to mediate the senescence of EPCs.

## Data Availability

The data that support the findings of this study are available from the corresponding author upon reasonable request.

## Supplementary Materials

Supplementary Materials Supplementary Table 1: detailed demographic and clinical characteristics of patients in this study.
