# Supplementary material for: p300/Sp1-Mediated High Expression of p16 Promotes Endothelial Progenitor Cell Senescence Leading to the Occurrence of Chronic Obstructive Pulmonary Disease
Source: Mediators Inflamm. 2021 Aug 19;2021:5599364. doi: 10.1155/2021/5599364 (PMC8397552; doi:10.1155/2021/5599364)
Supplement: Supplementary Materials — Supplementary Table 1: detailed demographic and clinical characteristics of patients in this study. [file 5599364.f1.docx]

non-smoking non-COPD:

n=18, Smoking (pack-years)=0, FEV1%pre=107.0±10.8, FEV1/FVC=83.9±6.8

smoking non-COPD：

n=20, Smoking (pack-years)=25.7±12.7, FEV1%pre=103.8±13.7, FEV1/FVC=79.0±4.4

smoking COPD：

n=20, Smoking (pack-years)= 37.3±13.0,FEV1%pre=43.2±6.9, FEV1/FVC=50.5±7.6

A: non-smoking non-COPD, B: smoking non-COPD, C: smoking COPD

**Patient information table S1**

| No | Gender | Age | BMI (kg/m^2^) | Smoking (pack-years) | FEV1%pre | FEV1/FVC% |
| --- | --- | --- | --- | --- | --- | --- |
| A1 | male | 61 | 20.9 | 0 | 114 | 91.5 |
| A2 | male | 60 | 22.64 | 0 | 110 | 87.1 |
| A3 | male | 63 | 24.32 | 0 | 96 | 77.4 |
| A4 | male | 58 | 18.99 | 0 | 101 | 76.2 |
| A5 | male | 70 | 26.32 | 0 | 98 | 78.7 |
| A6 | male | 72 | 24.51 | 0 | 102 | 79.9 |
| A7 | male | 70 | 23.11 | 0 | 111 | 80.5 |
| A8 | male | 58 | 24.15 | 0 | 90 | 86.8 |
| A9 | male | 59 | 19.56 | 0 | 108 | 81.5 |
| A10 | female | 62 | 19.88 | 0 | 118 | 92.1 |
| A11 | male | 46 | 21.88 | 0 | 109 | 82.0 |
| A12 | male | 51 | 23.17 | 0 | 92 | 75.4 |
| A13 | male | 56 | 25.12 | 0 | 122 | 92.9 |
| A14 | female | 54 | 19.84 | 0 | 107 | 80.3 |
| A15 | male | 48 | 19.67 | 0 | 124 | 93.4 |
| A16 | male | 57 | 22.67 | 0 | 121 | 91.0 |
| A17 | male | 47 | 25.32 | 0 | 112 | 90.8 |
| A18 | male | 53 | 22.34 | 0 | 91 | 73.0 |
| B1 | male | 56 | 20.18 | 24 | 96 | 76.1 |
| B2 | male | 42 | 23.22 | 8 | 115 | 77.5 |
| B3 | male | 53 | 19.89 | 25 | 92 | 82.6 |
| B4 | female | 57 | 20.45 | 15 | 113 | 81.1 |
| B5 | male | 48 | 24.21 | 6 | 125 | 88.1 |
| B6 | male | 67 | 23.34 | 45 | 86 | 72.3 |
| B7 | male | 72 | 20.19 | 36 | 84 | 72.7 |
| B8 | male | 63 | 20.86 | 29 | 110 | 80.2 |
| B9 | male | 75 | 21.56 | 49 | 83 | 74.0 |
| B10 | male | 65 | 23.76 | 17 | 123 | 85.9 |
| B11 | male | 68 | 25.13 | 20 | 97 | 74.5 |
| B12 | male | 50 | 18.67 | 14 | 101 | 78.8 |
| B13 | male | 48 | 19.34 | 27 | 95 | 76.6 |
| B14 | male | 45 | 20.56 | 16 | 124 | 84.3 |
| B15 | male | 56 | 28.13 | 30 | 114 | 82.0 |
| B16 | female | 60 | 20.45 | 28 | 102 | 77.2 |
| B17 | male | 55 | 22.21 | 10 | 107 | 79.7 |
| B18 | male | 75 | 22.34 | 47 | 116 | 83.4 |
| B19 | male | 62 | 20.19 | 33 | 107 | 78.3 |
| B20 | male | 71 | 22.12 | 35 | 85 | 75.1 |
| C1 | male | 68 | 21.66 | 50 | 32 | 35.4 |
| C2 | female | 59 | 21.56 | 24 | 36 | 45.5 |
| C3 | male | 55 | 19.89 | 18 | 48 | 53.8 |
| C4 | female | 57 | 20.45 | 21 | 37 | 43.2 |
| C5 | male | 58 | 24.21 | 40 | 52 | 60.7 |
| C6 | male | 57 | 23.34 | 22 | 43 | 46.6 |
| C7 | male | 62 | 20.19 | 39 | 46 | 48.3 |
| C8 | male | 74 | 20.86 | 56 | 45 | 52.7 |
| C9 | male | 55 | 21.56 | 26 | 49 | 55.8 |
| C10 | male | 65 | 23.76 | 42 | 47 | 50.8 |
| C11 | male | 73 | 25.13 | 55 | 44 | 47.2 |
| C12 | male | 50 | 18.67 | 20 | 50 | 56.4 |
| C13 | male | 60 | 19.34 | 41 | 56 | 62.4 |
| C14 | male | 53 | 20.56 | 39 | 35 | 42.7 |
| C15 | male | 56 | 28.13 | 32 | 40 | 59.4 |
| C16 | male | 63 | 20.45 | 43 | 39 | 58.5 |
| C17 | male | 65 | 22.21 | 27 | 51 | 57.8 |
| C18 | male | 58 | 22.34 | 38 | 38 | 44.5 |
| C19 | male | 74 | 20.19 | 54 | 32 | 38.6 |
| C20 | male | 79 | 22.12 | 58 | 43 | 49.7 |
